# Reconstruction of Fingers Using Skin-Bone Grafts with Microvascular Anastomoses

**DOI:** 10.17691/stm2020.12.1.02

**Published:** 2020

**Authors:** N.M. Aleksandrov, S.V. Petrov, D.A. Kuptsov, M.S. Petrov

**Affiliations:** Leading Researcher, Microsurgical Department, Institute of Traumatology and Orthopedics, Privolzhsky Research Medical University, 10/1 Minin and Pozharsky Square, Nizhny Novgorod, 603005, Russia; Leading Researcher, Microsurgical Department, Institute of Traumatology and Orthopedics, Privolzhsky Research Medical University, 10/1 Minin and Pozharsky Square, Nizhny Novgorod, 603005, Russia; Clinical Resident, Institute of Traumatology and Orthopedics, Privolzhsky Research Medical University, 10/1 Minin and Pozharsky Square, Nizhny Novgorod, 603005, Russia; Associate Professor, Department of Surgery, The University of Auckland, Private Bag 92019, Victoria St., Auckland, 1142, New Zealand

**Keywords:** stumps of fingers, segmental defects of the phalanges, metacarpal defects, microsurgery, transplantation of skin-bone grafts, transplantation of non-free skin-fat flaps

## Abstract

**The aim of the study** was to present a clinical and anatomical rationale for transplantation of skin-bone grafts with microvascular anastomoses for treating terminal and segmental defects of the hand and fingers.

**Materials and Methods:**

Finger and metacarpal bones were reconstructed in 25 hands of 25 patients by transplanting skin-bone tubular fragments with microvascular anastomoses. Transplants from the second metatarsal bone (n=22) and fibula (n=3) were used. Clinical, radiological, morphological, biomechanical, biophysical, and statistical research methods were used. The developed technology is adjustable to individual vascular anatomy of the foot. The proposed use of non-free skin-fat flaps and skin-bone fragments with microvascular anastomoses has been implemented for reconstructing lost segments.

**Results:**

The engraftment of skin-bone fragments was observed in 25 cases. In two cases, partial necrosis of the transplants was detected. Sufficient resistance of the transplanted bone graft to resorption was noted. According to the X-ray evidence, the length of the finger with the metacarpal bone after surgery was 8.44±0.32 cm, in the short term after surgery — 8.10±0.36 cm, and in the long term — 7.87±0.45 cm, indicating mild resorption. We used an individual approach to the transplant selection, which made it possible to obtain generally good long-term results in 3 patients, and satisfactory results — in 22 patients.

**Conclusion:**

The study showed the feasibility of transplanting skin-bone fragments with microvascular anastomoses for replacing various anatomical defects of the hand and fingers. The proposed modification takes into account the variability of vascular anatomy of the donor region.

## Introduction

At the current surgical practice of finger reconstruction, the second toe or part of the first toe on microvascular anastomoses are used together with relocation of a skin-bone radial flap on the peripheral vascular pedicle [1–5]. However, these interventions inevitably cause a defect in the donor area, which creates a number of difficult problems [1]. In this regard, alternative methods of finger reconstruction free of this disadvantage are required. Skin-bone transplants on microvascular anastomoses may be good candidates for this role because they have limited functional significance and their removal creates a minimal cosmetic defect in the donor area. These approaches are still rarely used. Isolated cases of transplantation of a bone fragment from the lateral edge of the scapula, iliac wing, metatarsal fragment, or tibia crest have been reported; however, these reports have little clinical and anatomical rationale [1–9]. In most cases, the authors do not have adequate clinical material and do not provide long-term treatment results. Moreover, these interventions were mostly based on using marginal cortical-spongy grafts of flat bones. The possibilities of using organotypic grafts from the diaphysis of tubular bones are practically not reported.

**The aim of the study** was to present a clinical and anatomical rationale for transplantation of skin-bone grafts with microvascular anastomoses for treating terminal and segmental defects of the hand and fingers.

## Materials and Methods

Finger and metacarpal bones were reconstructed in 25 hands of 25 patients by transplanting skin-bone tubular fragments with microvascular anastomoses. The patients were predominantly male (n=24) with the average age of 31.9±1.8 years.

The study was conducted in accordance with the Helsinki Declaration (2013) and approved by the Ethics Committee of the Privolzhsky Research Medical University. Informed consent was obtained from each patient.

The primary reconstruction (4 cases) was performed 10.6±6.8 h after injury, and the secondary operation (21) — 205.2±38.8 days later. The right hand was operated on in 16 cases, and the left — in 9 cases. Mechanical damage was the cause in 21 patients, and combined trauma (mechanical + burn) — in one case. In addition, there was one case associated with a gunshot injury, one — with electrocution, and one — with frostbite. Reconstruction of the first finger and metacarpal bone was carried out in 18 patients, the second finger and metacarpal bone — in 2 patients, and the ulnar edge of the hand — in 5 patients. In 19 cases, terminal defects of the fingers and metacarpal bones were repaired, and in 6 cases — segmental defects.

The donor material was taken from the second metatarsal bone (22) and fibula (3). Using a blood-supplied skin-bone graft from the second metatarsal bone, the following defects were repaired: terminal defects of the lost fingers (14), segmental defects of the metacarpal bones (2), and the main phalanx (1). In addition, the following surgical manipulations were performed: formation of the ulnar supportive jaw combined with interposition of non-vascular bone graft (2) repair of a segmental defect in the metacarpal bone of the restored finger in combination with simultaneous replacement of the damaged finger in the total absence of the radial edge of the hand (2), repair of a subtotal segmental metacarpal defect (1). A fibula graft was used to form the ulnar supportive jaw (3).

When repairing terminal defects and defects of the ulnar edge of the hand, we operated on finger stumps at the base of the main phalanx (3), the metacarpal head (7), the distal third of the metacarpal bone (2), the middle third of the metacarpal bone (2), as well as the complete absence of the first finger or the second to fifth fingers together with metacarpal bones (5). The following anatomical types of terminal defects of the fingers were noted [10]: type I — 6, type II — 2, type III — 5, type V — 1, type VII — 1, type VIII — 3, and type IX — 1.

To replace preexisting and newly formed defects of the hand soft tissues and restore the soft tissue skeleton, plastic surgery with non-free skin-fat flaps was used in most patients. For this purpose, a sharp Filatov stalk (2), a Filatov flap on two feeding pedicles (15), a double Blokhin–Converse flap (4), a double flap combined with a scapular skin-fat flap on microvascular anastomoses (1) were used for transplantation. Only in three cases of segmental replacement of skin-bone defects of the metacarpal bone and the main phalanx, additional plastic work was not required due to a small size of the soft tissue defects.

Osteosynthesis of bone fragments was carried out mainly by using pins (16). In addition, osteosynthesis was also performed by using pins in combination with the insertion method (9). Bone grafts were transplanted together with an islet skin-fat flap (a small “sentinel” one or that capable of replacing large defects in accordance with the type of the graft and the condition of the skin-septal vessels) with the formation, as a rule, of a muscular sleeve.

The fibula autograft was taken using the Taylor method, with the formation of a muscular sleeve of 5–6 mm thick according to the limited size of the recipient area on the hand. The size of the “sentinel” flap was limited by the anatomical pattern of the septal vessels, which varied from 3×5 to 5×7 cm. The anatomy of the peroneal artery and veins was more consistent; for this reason, the peroneal artery was always used as a feeding vessel. In all cases of using a transplant originated from the fibula, the venous outflow was provided by anastomoses from one of the veins accompanying the feeding artery.

For the purpose of creating an organotypic skin-bone graft from the dorsal pedal artery, including the second metatarsal bone, we developed a technology based on the individual vascular anatomy of that region. Before the operation (n=21), an ultrasound test was performed on the dorsal pedal vessels to identify the first dorsal metatarsal artery and measure its depth and diameter. Based on the results, a zigzag incision was made in the projection of this artery; the incision continued into the middle of the first intertarsal gap. The dorsal pedal artery was isolated and mobilized along with the deep veins and the deep branch of the peroneal nerve up to the ankle. At the same time, the tarsal branches of the artery were ligated and cut. The arched foot artery was ligated and cut at several locations depending on the anatomy of the first and second dorsal metatarsal arteries. If the first dorsal metatarsal artery alone was present (n=20), the arched artery was ligated at its exit from the dorsal pedal artery. In the presence of the second dorsal metatarsal artery (n=2), the arched artery was ligated and cut laterally to the previous one. This approach made it possible to include both arteries in the would-be tissue graft. In one patient, the second metatarsal artery was dominant, and in the other patient, it was the only one. Next, the plantar branch of the dorsal pedal artery was ligated and cut in the interval between the proximal sections of the first and second metatarsal bones. If the second dorsal metatarsal artery had a deep plantar branch, it was also ligated and cut.

In the projection of the first dorsal metatarsal artery, an oval-shaped “sentinel” skin-fat flap was formed while preserving its connections with the first dorsal metatarsal artery. To that end, in the area of the second metatarsal bone, a longitudinal arcuate incision was made; its ends were connected with the incision in the area of the first intertarsal gap. The first dorsal interosseous muscle was separated from the second metatarsal bone with a muscle sleeve 0.5–0.6 cm thick preserved on it. In the presence of the second metatarsal artery, the muscle sleeve was also formed along the lateral surface of the second metatarsal bone. A branchlet going from the first dorsal metatarsal artery to the first finger was ligated, cut, and mobilized on the medial surface, while preserving maximally the branches leading to the second metatarsal bone. The position of this artery differed in different patients. The first and second dorsal metatarsal arteries (if any), which essentially continue the dorsal pedal artery are ligated and cut in all cases distally to the formed skin-fat flap. A transverse osteotomy was performed in the area of the distal and proximal metaepiphyses of the second metatarsal bone, while preserving the connections between the formed fragment of the second metatarsal bone and the skin-fat flap, muscular sleeve, and dorsal metatarsal arteries. The plantar interosseous muscles were separated from the fragment of the second metatarsal bone.

The skin flap necessarily included the dorsal pedal saphenous veins, located in its projection; these vessels were cut distally to the flap and mobilized to the level of the articular fissure of the ankle. In all cases, the largest vein proximal to the flap was preserved and the venous branches flowing into it were ligated to create a single venous collector. The vein anatomy of the *dorsum pedis* also varied significantly between patients. According to the anatomy of the dorsal subcutaneous venous network, the draining venous collector was formed from the main incision based on the central dorsal saphenous vein (15) or the medial saphenous pedal vein; in the latter case, an additional incision was made along the medial edge of the foot (6). In addition, the largest of the deep veins accompanying the dorsal pedal artery was used (1). If a subcutaneous venous pedicle was used for the venous drainage, then the veins accompanying the dorsal artery were ligated.

The developed microsurgical technology made it possible to form an adequately blood-supplied complex of tissues for various types of vascular anatomy of the foot. The presence of a pronounced first dorsal metatarsal artery, regardless of its location, in all cases provided the adequate blood supply to the skin flap and the entire tissue complex. After stopping vascular spasm, stabilizing blood circulation in the mobilized tissue fragment, and achieving hyperperfusion and adequate venous outflow (which was verified by pulsation of the arterial pedicle, the distal end of the first dorsal metatarsal artery and filling of the draining vein), the venous and arterial pedicles were cut along with the deep branch of the peroneal nerve. In the recipient area, the ends of the bone fragments were refreshed, the artery, vein, and nerve of the receptive bed were isolated. Next, the pedal tissue fragment was transferred to the hand and blood flow was restored in it by microvascular anastomoses from the arterial and venous pedicles to the radial or ulnar artery and the main vein. The veins accompanying the dorsal pedal artery were ligated if a subcutaneous vein pedicle was used for venous drainage. Most of the soft tissues were restored using the previously transplanted non-free skin-fat flaps. On the formed finger, the skin flap was placed on the working surface (terminal and palmar-ulnar) and innervated by suturing end-to-end the proximal end of the deep peroneal nerve and the surface branch of the radial nerve on the finger stump (3 cases). This approach proved useful for the subsequent improvement of finger functions and resistance to mechanical stress.

The adequate blood supply to the skin component of the graft indicated sufficient blood supply to the bone component of the graft as well. The width of the “sentinel” flap depended on the size of displacement of the dorsal pedal skin. To determine this size, the skin was folded in the dorsal area of the first intertarsal gap and the folds were marked out. The distance between the marks after the skin release indicates the required flap width, which (in our observations) did not exceed 3 cm, and its length was not limited. By using this approach, we were able to primarily suture the surgical wound in the donor area in all cases. Thus, the postoperative scar was normotrophic and its width did not exceed 0.1 cm. Earlier, this technique was proposed for reconstructing the first finger with total defects of the radial edge of the hand [11]; it allows one to restore an adequate length of the first finger using the distracting elongation of the transplanted fragment of the second metatarsal bone.

In the present study, clinical, radiological, morphological, biomechanical, and biophysical methods were used. Doppler ultrasonography was applied to examine the pedal vessels. For this purpose, we used an ACUSON X300 instrument (Siemens, Germany) with a 199 dB dynamic range and a 2D color tissue Doppler imaging option. The material resulted from the corrective surgery on the bone and soft tissues of the restored finger was subjected to morphological examination after hematoxylin and eosin staining,

The Statistica 6.0 and Statistica 10.0 software were used to process the primary data.

## Results

Soon after the finger reconstruction was performed using the above technology, partial necrosis of the interpositional (1 case) and terminal (1 case) bone grafts was detected; in our view, this complication did not significantly affect the long-term results. All non-free skin-fat flaps that had been transplanted took root. The donor wounds on the foot and lower leg got healed by primary tension with the formation of a linear scar, which did not cause any functional and cosmetic problems to the patient. Full grip of the hand was restored in 24 patients, partial grip — in 1 patient.

The long-term results in the period from 1 to 8.3 years were assessed by our modification of the Belousov method [12]. The following scores of biomechanical indicators were observed: adduction of the first finger — 4.67±0.26; abduction of the first finger — 4.13±0.35; opposition of the first finger — 3.63±0.63; flexion of the metacarpophalangeal joint — 1.0±0.0; palmar abduction — 4.33±0.33; radial abduction — 3.33±0.88. According to the assessment, 3 patients showed good results and 22 patients — satisfactory results. In patients with an innervated islet flap, the discriminatory sensitivity of the flap was 8–10 mm. The proposed transplantation of the skin-bone fragments on microvascular anastomoses was found more beneficial for isolated defects of the first finger as compared with combined defects: the number of good results was significantly greater (p=0.018). The results of the primary and secondary reconstructions did not significantly differ (р=0.12–0.69).

The X-ray studies showed no signs of sizable transplant resorption. We found that just after surgery, the finger length along with the metacarpal bone was 8.44±0.32 cm, in the short term after surgery — 8.10±0.36 cm, and in the long term — 7.87±0.45 cm, which indicates mild resorption. The width of the distal end of the graft after the operation was 0.92±0.07 cm, in the short term — 0.90±0.07 cm, and in the long term — 0.82±0.08 cm. A significant decrease (by 0.5–0.6 cm) in the length was noted for metacarpal bone grafts; these changes reached statistical significance when compared between the postoperative measurements and the short time results (p=0.007), or the long term results (p=0.005), as well as between the short and long-term results (p=0.03). In addition, there was also a statistically significant decrease in the width of the distal end of the graft (about 0.1 cm) when compared to the instant postoperative data with those of the distant period (p=0.004), as well as between the near and distant periods (p=0.02). In the long term, the graft’s end acquires a round shape, which may be associated with tissue devitalization in the osteotomy zone during graft collection. These structural changes did not significantly affect the treatment outcomes.

According to our results, a transplant from the second metatarsal bone can be taken with a maximum length of 5.0 cm while maintaining the bone head and base, which helps prevent retraction of the second radial pedal bone and deviation of the first and third toes. In the X-ray images, a bone marrow canal, internal and external cortical plates of the tubular graft and its normal structure and density, not differing from the patient’s own hand bones, are clearly identified; these results indirectly indicate the preservation of adequate blood supply to the graft. All transplants got consolidated within the time typical for the primary consolidation of metacarpal bones and phalanges. The formation of a bone regenerate during distraction of the transplanted segment also indicated its viability, as did the consolidation of interpositional vascular bone autografts.

**Figure 1:**
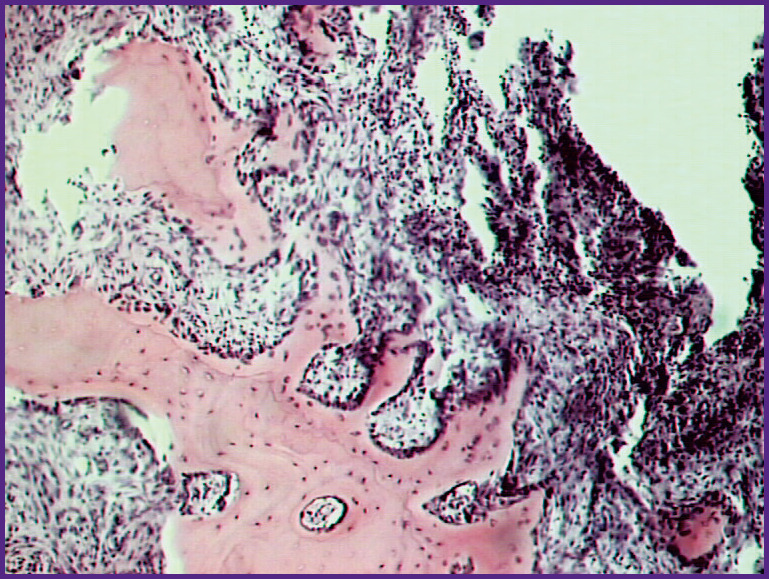
**Histological presentation of the transplanted fragment from the metatarsal bone in patient K., 37 years old:** newly formed diverse bone regenerates at the edges of the bone marrow spaces, cellular vascularized fibroreticular tissue; hematoxylin and eosin staining; ×135

The successful engraftment was also confirmed by the histomorphological findings in the transplanted fragments of the metatarsal bones (Figure 1). Thus, against the background of preserved bone structures with various manifestations of atrophy, necrobiosis, or complete death of bone tissue, there was consistent evidence of reparative proliferation. In a compact bone, they are manifested as restructuring and regeneration of osteons with restoration of blood flow in Haversian canals. In the metaepiphyseal areas of the graft, reparative processes are evidenced by the occurrence and development of plate regenerates at the edges of preserved or damaged cancellous bone beams. In the areas of their most complete development, they form spongy structures that contain remnants of preexisting or newly formed blood-supplying fibroreticular tissue in their bone marrow spaces with no significant signs of hemocirculatory disorders.

Thus, the use of tubular grafts from the second metatarsal bone provides organotypic restoration of phalanges and metacarpals with adequate functional and cosmetic outcomes.

**Figure 2:**
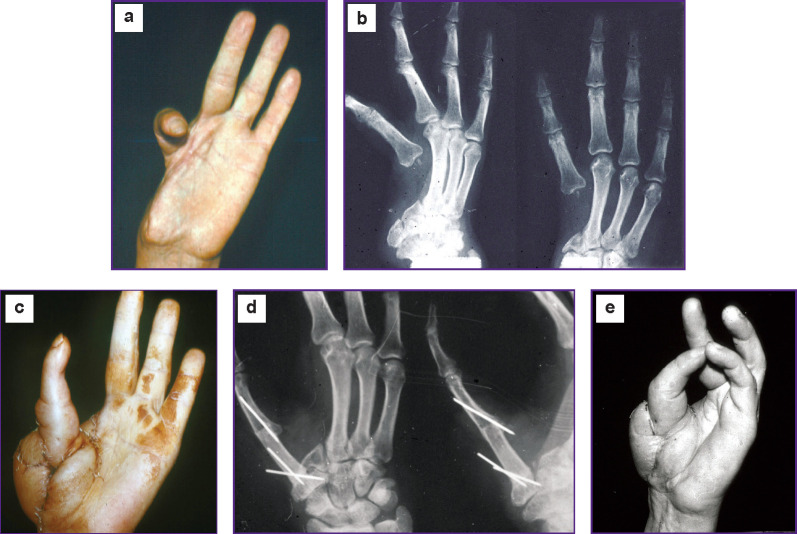
Treatment outcome in patient S., 43 years old, with a defect in the radial edge of the hand one year after transplantation: (a) the hand appearance before surgery; (b) X-ray images of the hand before surgery; (c) the hand appearance after plastic surgery on its radial edge, an accustomed sentinel flap can be seen; (d) X-ray images of the hand one year after repositioning the second finger and transplanting a fragment from the second metatarsal bone; a bone marrow canal and complete consolidation can be identified; (e) the opposing function of the first finger

Here, we present a clinical example of reconstruction of the first finger using a transplant from the second metatarsal bone (Figure 2).

*Patient S., 43 years old, was admitted to the clinic due to a total defect of his hand radial region, a defect of the second metacarpal bone, and extensor tendons of the second left finger after a severe mechanical injury.*

*Successful reconstruction of the radial edge of his hand was achieved by repositioning the second finger, transplanting the Filatov stalk and skin-bone graft from the second metatarsal bone. The grip over the hand has been restored.*

Some unsuccessful results (partial resorption of the graft bone) were observed in two cases with atypical vascular architectonics in the dorsal pedal area: an insufficient diameter of the dorsal metatarsal artery (less than 0.5 mm) and its complete absence (the plantar metatarsal artery was then used for anastomoses, instead).

## Discussion

In this work, we examined the possibility of transplanting tissue fragments from the second metatarsal bone and the fibula to segmentally damaged fingers. Modern microsurgical technologies allow for repairing various skin-bone defects by using multicomponent tissue fragments [13]. However, after taking such fragments from the fibula or second metatarsal bone, it becomes necessary to perform additional skin surgery on the donor region to replace the removed skin-bone fragment. The process of preparing and removing such a flap from the foot also leads to disturbance in toe sensitivity and exposure of their extensor tendons. This situation necessitates using an additional free skin flap to be transplanted into the donor area defect; in most such cases, the new pedal transplant does not take root, it forms scars by combining with tendons, and eventually causes extensor contractures of the toes; in addition, it often gets injured by shoes, becomes hyperpigmented and ulcerated [1]. To avoid this drawback, some authors transplanted a primary skin-fat flap with microvascular anastomoses or repositioned it together with a vascular pedicle to a soft tissue defect on the pedal dorsum [1, 14]. However, this approach prolongs the time of surgery and makes it more invasive [1]. In addition, simultaneous transplantation of several tissue fragments with microvascular anastomoses is a labor-consuming procedure associated with numerous complications. A skin-fat flap transplanted to the donor area on the pedal dorsum is not sensitive at all; it requires additional corrective operations, thus significantly prolonging the treatment period. Perhaps, for this reason, transplantation of unipolar grafts from the second metatarsal bone did not find wide application in reconstructing terminal defects of the fingers. In any case, the minimization of the donor area damage is mandatory for any innovative technique in autotransplantation [1].

Since 1996, the principle of minimal surgical aggression has been used in our microsurgery clinic for the collection of donor autograft [15, 16]. The proposed approach includes preliminary plastic surgery on the soft tissues of the recipient area, the formation of the soft tissue skeleton on the finger using non-free skin-fat flaps, and transplantation of a graft from the second metatarsal bone or fibula with microvascular anastomoses together with a small sentinel skin-fat flap. In this approach, hyperperfusion of the transplanted graft and an improvement in its viability are achieved. In addition, this approach allows one to close the surgical wound in the donor area using the surrounding tissues, which minimizes cosmetic and functional defects, reduces the duration and invasiveness of the intervention and maintains the sensitivity of the foot dorsal skin, although it leads to a slight increase in the duration of treatment.

The developed approach is beneficial from the perspective of variable vascular anatomy of the foot. Our clinical observations showed that the first dorsal metatarsal artery is the dominant route of blood supply to the transplant; this vessel was identified in 88.4% of patients and had a sufficiently large diameter in 92.1% of those cases. If the second dorsal metatarsal artery is reliably identified, it should also be incorporated in the tissue graft. If the first dorsal metatarsal artery is hardly detectable, has a diameter of <0.5 mm, or completely absent, and the second metatarsal artery is not found, no adequate blood supply to the transplant is ever achievable. In our opinion, under such conditions, surgery is contraindicated. This notion corroborates with study of Borovikov [1], who concluded that if the vessels of the first intertarsal gap are <0.5 mm in diameter, transplantation of foot tissue fragments is contraindicated. For this reason, it is obligatory (in our view) to conduct a preoperative vascular ultrasound examination of the dorsal foot surface and the first tarsal gap to assess the size and shape of the blood vessels.

Our results indicate that blood-supplied skin-bone tubular grafts on microvascular anastomoses can be successfully used for finger reconstruction along with and in combination with other treatment methods. When using a tubular transplant, its endosteal and periosteal vessels with all connections between them are preserved; consequently, the circulation typical of an intact bone is preserved as well. Under the consistent tissue hyperperfusion and circulation in a closed vascular network, blood is supplied to all compartments of the transplant and thus provides for a good resistance to resorption. If an edge transplant is formed from a cancellous bone, it has no endosteum; a transplant from a tubular bone does have the periosteum and endosteum but within a limited area, which prevents the organotypic transformation of the graft. By their architecture, size, and structure, transplants from the second metatarsal bone are most similar to the metacarpal bones and phalanges of the hand, which makes it possible to obtain satisfactory anatomical and functional outcomes.

The choice of transplant must be personalized according to the nature of the hand defect and the level of finger amputation. In our opinion, skin-bone reconstruction of the finger is especially indicated if the patient is willing to preserve the normal number of toes. The use of fibula grafts for total defects of the ulnar edge of the hand allows for the formation of an adequate ulnar supporting jaw of the required length, which naturally increases the gripping force and improves the ability to hold large and heavy objects [17]. A limitation of this method is its inability to recreate a finger having interphalangeal and metacarpophalangeal joints. This disadvantage is leveled by transplanting a graft onto the stump of the main phalanx, if its metacarpophalangeal joint is mobile. In this case, a newly formed finger can move this joint. Strong indications for such an operation, in our opinion, also arise in cases when the patient is reluctant to use the donor resources of the damaged hand or the forearm in reconstructing surgery for a missing finger. In the case of a segmental defect of the main phalanx or metacarpal bone, the indication for the use of the method is absolute. Under these conditions, it is possible to restore movements in the joints of the reconstructed finger.

## Conclusion

The present clinical and anatomical results show the feasibility of using the developed technology for the transplantation of skin-bone tubular grafts with microvascular anastomoses to repair defects of the hand and fingers. Transplants from the second metatarsal bone have clinical and anatomical advantages because they practically do not undergo changes during transplantation.

Our approach allowed us to minimize defects in the donor area after collecting a tubular bone graft for subsequent transplantation. When choosing and creating a transplant, the clinical and anatomical features of the donor and recipient areas should be taken into account.
